# Development of a high-resolution multiplex qPCR method to profile microbial consortia in spaceflight water recovery systems

**DOI:** 10.1016/j.bioflm.2026.100349

**Published:** 2026-01-29

**Authors:** Amber Dowell Busboom, Jiseon Yang, Taylor M. Ranson, Cheryl A. Nickerson, Evan G. Ortiz, Robert J.C. McLean

**Affiliations:** a Department of Biology, Texas State University, 601 University Drive, San Marcos, TX, 78666, USA; b Biodesign Center for Fundamental and Applied Microbiomics: Arizona State University, Tempe, AZ, USA; c School of Life Science: Arizona State University, Tempe, AZ, USA

**Keywords:** Life support, Bacterial quantification, Biofilm, Water recycling, Polymicrobial, Spaceflight

## Abstract

A key component of life support on the International Space Station (ISS) is the Water Recovery System (WRS), which recycles and disinfects urine, other wastewater, and humidity condensate for use as potable water. A resident mixed-species bacterial population has persisted in the WRS, upstream from the disinfection components, despite various microbial control methods. Five bacterial species (*Pseudomonas aeruginosa, Burkholderia contaminans*, *Methylobacterium fujisawaense, Ralstonia insidiosa,* and *Cupriavidus metallidurans*) have been regularly isolated from the WRS and have a propensity to form biofilms, which can reduce susceptibility to antimicrobial treatments. WRS organisms have been associated with biofouling and potential corrosion of valves and filtration components. Currently, microbial monitoring on the ISS requires samples to be collected and sent back to Earth for culture-based analysis, a process that can take months, given the constraints of spacecraft scheduling. Culture-independent monitoring could be established on the ISS and other spacecraft, thereby enabling rapid estimates of microbial population density and individual species’ susceptibility to disinfection. This technology will be critical in future missions beyond low Earth orbit, when access to reference labs is not possible. Here, we report on the development and validation of a multiplex quantitative Polymerase Chain Reaction (qPCR) method to identify and quantify members of a five species model biofilm community acquired from the ISS. Species-specific primers and probes detect organisms to an estimated detection limit of 10^4^–10^6^ CFU and detect the specific targeting of *C. metallidurans* by ampicillin when overall population levels were not significantly changed.

## 1 Introduction

Polymicrobial communities are ubiquitous on Earth and have traveled with humans into space [1]. Effective monitoring of microbial communities in closed environments such as the International Space Station (ISS) is essential for maintaining system functionality and ensuring crew health. Culture-based microbial monitoring protocols were established during construction of the ISS [2] and require microbial sampling by space crew, transport to Earth on scheduled resupply spacecraft, and delivery to a reference laboratory for analysis. The logistics of this process mean that weeks or even months can transpire between sample collection and analysis, and quantitative microbial population studies are not feasible. Culture-independent protocols for microbial identification have been conducted on the ISS, including nanopore-based sequencing and genome assembly [3,4], coupled with validation of microbial identification in a reference lab [5,6]. Quantitative polymerase chain reaction (qPCR) is being used as a rapid culture-independent technique to evaluate microbial populations including those that have been isolated from the ISS [7]. However, to date most qPCR investigations of spacecraft organisms have been performed after the samples were returned to Earth [7]. As proof of concept, a field based semi quantitative PCR kit was evaluated for specificity, on the ISS that targeted extracted genomic DNA from *Pseudomonas aeruginosa* [6] during flight operations. In this study, ground-based evaluations including genomic extraction and qPCR measurements were done from cultures of *P. aeruginosa, Aeromonas hydrophilia, Escherichia coli*, and *Salmonella enterica* var. Typhimurium, as well as sampling *S.* Typhimurium that had been placed on a tomato surface as a representative food item. The authors found no significant differences between the critical threshold level (Cq) of three dilutions of *P. aeruginosa* genomic DNA samples in qPCR conducted during flight, when compared to tests performed in a ground-based laboratory [6].

Onboard the ISS, the Environmental Control and Life Support System (ECLSS) controls atmospheric pressure, oxygen levels, water supply, waste management, and fire detection and suppression. The Water Recovery System (WRS) ECLSS component collects, recycles, purifies and disinfects humidity condensate and urine, creating potable water for astronauts [8]. Maintaining the WRS is critically important for successful long-term space flights beyond low Earth orbit where supplies cannot be readily accessed or renewed. One of the complications that has arisen on multiple occasions is the clogging of WRS waterlines by biofilm-containing biomass [9]. In addition to clogging waterlines, biofilms have been associated with microbial induced corrosion and can represent a potential reservoir of harmful bacteria [8]. Continual microbial monitoring has been conducted on the ISS including various ECLSS components [2] and many isolates have been characterized and preserved in NASA collections. While many bacteria have been isolated from the WRS (reviewed in Ref. [1]), a five-species frequently isolated consortium consisting of *Pseudomonas aeruginosa, Burkholderia contaminans*, *Methylobacterium fujisawaense, Ralstonia insidiosa,* and *Cupriavidus metallidurans* have been selected to investigate WRS biofouling mitigation protocols and these organisms have been fully sequenced [10]. During any biofilm mitigation studies, it will be important to know whether treatments target all organisms or are restricted in scope. Several ground based studies, using culture-based analyses, are currently underway to control WRS biofilm formation. In one of these studies [11], three different strategies (phosphorus removal, use of AgF as a disinfectant, and a commercial Sher-Loxane® coating (Sherwin-Williams, Cleveland OH)) were employed to control mixed culture biofilms on Inconel® (a nickel-chromium allow). During this study, cultures were grown in an ersatz medium that resembles WRS liquid. Treatment with one or two of the control strategies resulted in population shifts in which different species reacted to the various treatments and in some cases biofilm and planktonic culture results differed. The authors found that only the combination of all three control measures (phosphorous removal, AgF disinfection, and the presence of the commercial coating) was effective in biofilm prevention during the experimental conditions used. Should a similar biofilm control protocol be performed during spaceflight, it would be essential to evaluate population changes.

Building upon the sequencing information [10], we developed a fast and practical approach for the accurate detection and quantification of a five-membered microbial consortium composed of biofilm-forming species commonly isolated from the WRS. We first identified genes unique to each of the five bacterial species and selected species-specific targets for assay design: *phzM* gene for *P. aeruginosa*, *efeB* gene for *B. contaminans*, *blaOXA* gene for *R. insidiosa, cnrC* gene for *C. metallidurans,* and *moxJ* gene for *M. fujisawaense*. Using these targets, we established a qPCR method for rapid and precise detection and quantification, well-suited for routine monitoring by crew members and timely intervention in closed environments such as the ISS. Here, we report a new targeted qPCR-based approach that provides a rapid and specific tool for tracking these organisms and supports improved strategies for controlling biofilm formation, critical for maintaining system integrity and microbial safety. Also, we demonstrate that this assay will identify the specific reduction of one ampicillin-susceptible community member, but not the other ampicillin-resistant members of the mixed population, when exposed to this antibiotic.

## 2 Materials and methods

### 2.1 Bacterial cultures

Five bacterial ISS isolates, whose whole genomes had been previously sequenced and published [10], were obtained from NASA Johnson Space Center (JSC): *P*. *aeruginosa* NASA JSC 0201761-1; *B*. *contaminans* NASA JSC 172630038-1; *M. fujisawaense* NASA JSC 092160098-2; *R*. *insidiosa* NASA JSC 171870003-1; and *C. metallidurans* NASA JSC 16243002-4. All strains could be grown on R2A agar or broth at 22 °C (the ambient temperature of the International Space Station (ISS) cabin environment [8]). Frozen stocks were stored at −80^o^C with glycerol (12.5 % v/v) added as a cryoprotectant. For culture revival, cells were aseptically scraped from the frozen cultures, streaked onto R2A agar plates, and following 48–72h growth at 22^o^C, single colonies were subsequently transferred into 5-mL of R2A broth. Cultures were incubated for 72 h at 22 °C, with shaking at 200 rpm in a water bath. Colony-forming units (CFU/mL), determined by serial dilution and plating, along with optical density measurements at 600 nm (OD_600_), are shown in Fig. S1.

### 2.2 Comparative genomic analysis, functional annotation and identification of unique genes

Whole genome sequences of the five ISS isolates (*P*. *aeruginosa* (RefSeq: GCA_028961985.1), *C*. *metallidurans* (RefSeq: GCA_028920765.1), *R*. *insidiosa* (RefSeq: GCA_028920815.1), *B*. *contaminans* (RefSeq: GCA_028920775.1), and *Methylobacterium* sp. (RefSeq: GCA_028920805.1) were downloaded from the NCBI database (https://www.ncbi.nlm.nih.gov/nuccore/JAQPZM000000000). For each strain, both protein sequence files (.faa) and genome annotation files (.gff) were obtained. Protein FASTA files were uploaded to OrthoVenn3 [12] to perform ortholog clustering and identify shared and species-specific orthologous gene clusters. The resulting Orthogroups.txt file was used to extract protein IDs unique to each species. To functionally annotate these unique proteins, the corresponding.gff files were parsed using custom scripts to map protein IDs to their associated gene names, product descriptions, locus tags, and Gene Ontology (GO) terms. GO enrichment analyses were performed on species-specific ortholog clusters using standard GO annotation pipelines to identify enriched biological processes, molecular functions, and cellular components. To confirm the specificity of selected genes, we conducted nucleotide-level sequence alignments against the WGSs of all five species using SnapGene software (version 8.1.0) as well as into NCBI BLAST to validate their presence or absence across genomes.

### 2.3 Genomic DNA extraction

3 mL of broth culture from each strain was harvested by centrifugation and the genomic DNA was extracted using the DNeasy Blood & Tissue Kit (Qiagen), following the manufacturer's protocol for cultured cells. DNA concentration and purity were assessed using a NanoDrop spectrophotometer.

### 2.4 Species-specific Primer design

Species-specific primers were designed for the identified unique genes of each bacterial strain using NCBI Primer-BLAST. The nucleotide sequences of the unique genes (*phzM*, *blaOXA*, *cnrC*, *efeB*, and *moxJ*) were used as input templates. Primer parameters selected were: 1) primer length of 18–24 bp, 2) GC content of 50–60 %, 3) melting temperature (Tm) between 55 and 65 °C with ≤5 °C difference between primer pairs, and 4) a GC clamp at the 3′ end to enhance binding stability. Amplicon sizes were constrained to 100–150 bp to support downstream qPCR validation. To ensure specificity, primers were checked against the complete genome assemblies of all five species using Primer-BLAST's specificity filter, minimizing potential cross-reactivity. The candidate primer pairs were further tested using OligoAnalyzer (IDT Inc. Coralville IA) to screen for hairpin structures, self-dimers, hetero-dimers, and Tm mismatches. The top three primer sets per organism were then tested in silico using SnapGene to simulate PCR amplification and assess amplicon specificity, and then by amplification specificity using genomic DNA from each organism. The final primers selected after testing are listed in Table 1.

**Table 1:** Bacteria, Gene, Primer name, Primer sequence (5’ – 3’). Melting temperatures (Tm) were obtained from supplier (IDT, Coralville IA).

Bacterial species	Gene	Primer Name	Tm ^o^C	Sequence (5’ – 3′)	Amplicon size (bp)
*Burkholderia contaminans*	*efeB*	BC2–F	60.1	AGTTCCGTGTTGTCCCAGTGC	169
		BC2-R	59.5	ACCTGATGGGCTTCAAGGACG	

*Cupriavidus metallidurans*	*cnrC*	CM3-F	58.0	CCAATCGGCCATATCCCAGG	137
		CM3-R	56.9	GCAATTCCATCAGTGGCGTC	

*Methylobacterium fujisawaense*	*moxJ*	MF2-F	59.3	CTGACCACGAAGCCGTACTACC	154
		MF2-R	59.5	TATCCTTGAGCATCGCCTCGC	

*Pseudomonas aeruginosa*	*phzM*	PA2-F	59.2	TCGGATACATCAGGTGGGCG	172
		PA2-R	59.5	GACGTGTTGCCGTTCTTCGC	

*Ralstonia insidiosa*	*blaOXA*	RI1-F	59.6	CAATTCACGGCAAGACGGGC	159
		RI1-R	59.9	GCTGCATTTGTGCGTCCTGG	

### 2.5 PCR and electrophoresis

Single plex PCR was performed using GoTaq™ Green Master Mix (Promega) in a 25 μL reaction containing 12.5 μL master mix, 0.5 μL each of 10 mM forward and reverse primers, 1 μL DNA template, and 10.5 μL nuclease-free water. The optimized PCR conditions consisted of initial denaturation, 95^o^C 2 min; 30 cycles of 95^o^C denaturation 30 s, annealing 54^o^C for 15 s, and extension 72^o^C for 60 s; followed by a final extension of 72^o^C for 5 min. The PCR products were visualized by 1 % agarose gel electrophoresis (0.6 g agarose in 60 mL 1 × TAE with Gel Red (Sigma-Aldrich)). Five microliters of PCR product along with 1-μl 6X loading dye (New England labs) were loaded into each lane, and a 100 bp DNA ladder (Thermo Fisher) was also loaded as a reference. Gels were run at 80 V for 40 min and imaged using a Bio-Rad GelDoc™ analyzer.

### 2.6 Design of qPCR probes

The species-specific primer sets, and corresponding amplicon sequences were imported into PrimerExpress™ 3.0 software to design TaqMan™ probes (Table 2). TaqMan chemistry was selected to enhance sequence specificity, which is critical for distinguishing targets in multiplex assays [13]. Fluorescent dyes were assigned based on expected target abundance by pairing the brightest dye with the lowest abundance target. *P. aeruginosa* was assigned FAM dye, *B. contaminans* and *R. insidiosa* were assigned VIC dye, and *M. fujisawaense* and *C. metallidurans* were assigned ABY dye. ROX was chosen as the reporter dye.

**Table 2:**
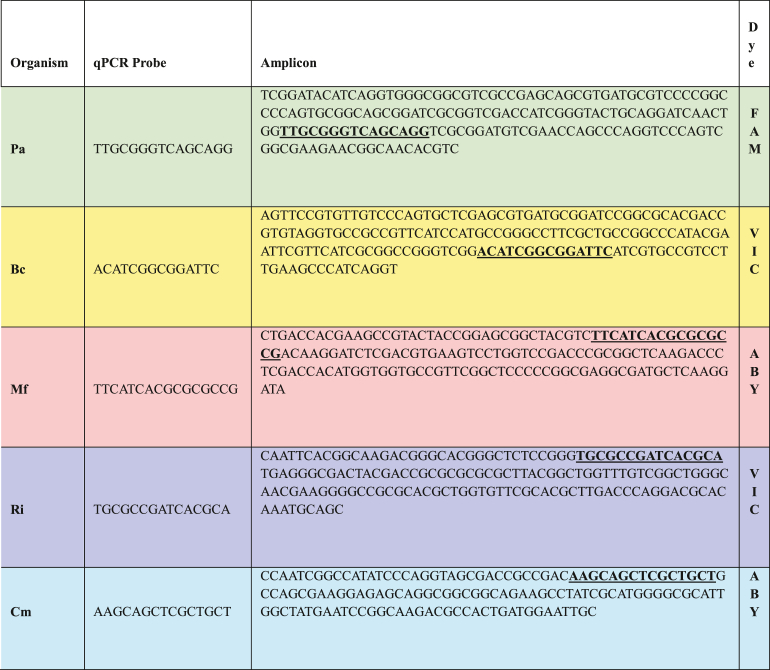
Amplicons showing TaqMan™ probe sequences (bold, underlined), location, and dyes (ABY – Applied Biosystems ABY™ dye; FAM - Fluorescein amidites 6-carboxyfluorescein; ROX – 6-carboxy-X-rhodamine dye; VIC – 2′-chloro-7′-phenyl-1,4-dichloro-6-carboxyfluorescein). Organisms are designated as Bc – *Burkholderia contaminans*; *Cm* – *Cupriavidus metallidurans*; Mf – *Methylobacterium fujisawaense*; Pa – *Pseudomonas aeruginosa*; and Ri – *Ralstonia insidiosa*.

### 2.7 Single and multiplex qPCR set up

Single plex qPCR reactions were performed using DNA extracted from pure cultures as described above. Serial dilutions for each template were prepared to evaluate qPCR detection limits and specificity. For multiplex PCR, Each 20 μL reaction contained 10 μL TaqMan™ Master Mix (2X), 1 μL TaqMan™ Assay (20X), 7 μL nuclease-free water, and 2 μL of DNA template. Reactions were run in triplicate, including no-template controls. Amplification was carried out on a QuantStudio 3 instrument (Thermo Fisher) using the Fast Comparative Ct – TaqMan™ protocol: 95 °C for 20 s (enzyme activation), followed by 40 cycles of 95 °C for 1 s and 60 °C for 20 s. Standard curves were generated to assess PCR efficiency.

### 2.8 Assessment in mixed culture

To test the qPCR protocol in a mixed culture setting, four of the cultures: *B*. *contaminans*, *C. metallidurans, P*. *aeruginosa,* and *R. insidiosa* were revived from frozen stock on LB agar (Lennox recipe). *M. fujisawaense* was not used in this test to serve as a negative control. Prior to experimentation, the organisms were tested for growth in the presence of several antibiotics as well as AgF. Our rationale was to test general planktonic and biofilm growth on one type of media to provide background values, and then under a treatment that would inhibit a subset of these four organisms. *C. metallidurans* did not grow on LB containing 100 μg/ml ampicillin, whereas *B*. *contaminans*, *P*. *aeruginosa,* and *R. insidiosa* did grow (Fig. S2), so ampicillin was selected as the antimicrobial treatment. We used a similar protocol for biofilm and planktonic cultures as described in Ranson et al. [14]. Starter cultures were prepared by transferring isolated colonies of the four test organisms to test tubes containing 10 ml LB and incubated overnight in a shaking water bath at 30^o^C, 200 rpm. The starter cultures were used to inoculate (100 μl per culture) into 50-ml flasks (n = 3 for each condition) each containing 25 ml LB or LB plus ampicillin (100 μg/ml) and three silicon rubber disks as biofilm colonization substrata. The culture flasks then incubated for 48h at 30^o^C, 200 rpm. Following incubation, planktonic and biofilm cultures were collected and population levels measured by dilution plating for planktonic cultures or sonication followed by dilution plating [14]. DNA extracts from planktonic cultures were obtained with the extraction protocol described above. For biofilm populations, sonicated cells in PBS from all 3 silicon rubber disks in each culture vessel, were concentrated by centrifugation (14,600×*g* for 5 min) and then extracted using the DNeasy kit (Qiagen) following the manufacturer's instructions.

## 3 Results and discussion

### 3.1 Developing species-specific primer sets for 5 strains and verifying cross-species specificity

**Fig. 1:**
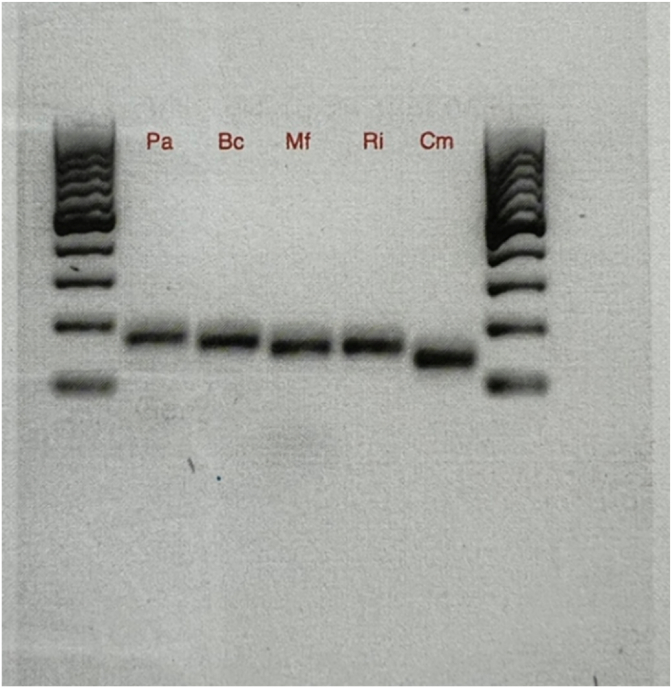
– Following PCR optimization, the species-specific primers amplify the target DNA at the expected product size in single plex PCR. A 100 bp DNA ladder (Thermoscientific 100 bp GeneRuler^Tm^) is shown in the two outside lanes with the two bottom bands being 100 and 200 bp respectively.

Species-specific primer sets were designed as described in the Methods section and screened for specificity using PCR simulations in Snapgene. Based on predicted specificity and performance, one primer set per strain was selected for experimental validation (Table 1). As shown in supplementary information, each selected primer set was tested by PCR using genomic DNA from all five bacterial strains to evaluate cross-species specificity. As described in the supplemental information the annealing temperature was increased and extension time reduced during PCR optimization to improve specificity. These adjustments successfully resulted in specific PCR products at the expected size (Fig. 1).

### 3.2 Species-specific qPCR probe design and sensitivity evaluation

**Fig. 2:**
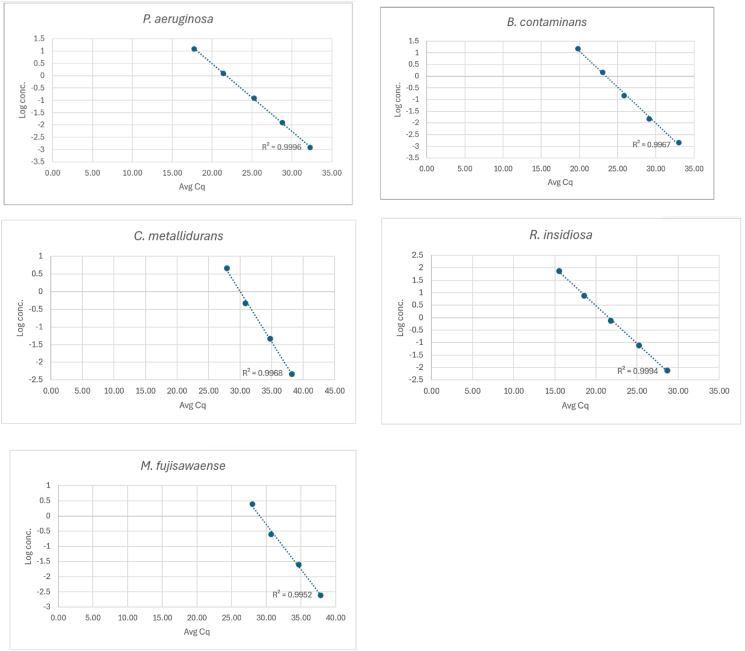
– qPCR amplification plot of ten-fold dilutions of *P. aeruginosa* with a starting concentration of 12 ng/μl, *B. contaminans* starting concentration of 15 ng/μl, *M. fujisawaense* with a starting concentration of 2.5 ng/μL, *R. insidiosa* with a starting concentration of 77 ng/μL, and *C. metallidurans* with a starting concentration of 4.7 ng/μL. The correlation coefficients are shown. Calculated PCR efficiencies are: *P. aeruginosa* 88.31 %, *B. contaminans* 102.8 %, *M. fujisawaense* 98.4 %, *R. insidiosa* 101.3 %, and *C. metallidurans* 93.9 %.

Species-specific TaqMan™ qPCR probes were designed as described previously and the qPCR probes and the amplicon sequences are shown in Table 2. We validated using single plex reactions to determine the detection range, sensitivity and PCR efficiency for each primer set. Serial 10-fold dilutions of genomic DNA were prepared for each organism, spanning the following concentration ranges (ng/μL): *P. aeruginosa* (0.0012–12.5), *B. contaminans* (0.0015–15), *M. fujisawaense* (0.00025–2.5), *R. insidiosa* (0.0077–77), and *C. metallidurans* (0.00047–4.7). Standard curves were generated for each primer-probe set, and the coefficient of determination (R^2^) exceeded 0.99 for all assays, indicating excellent linearity and dynamic range (Fig. 2). PCR efficiency is shown in the legend for Fig. 2. The values of the DNA yield and purity, culture data from which the DNA was extracted, the minimum DNA detected and the corresponding estimate of the minimum CFU detected are shown in Table 3.

**Table 3:** DNA extraction, purity, limits of detection, estimated Cq in single plex and triplex conditions. Cq values are based on readings of 10 ng/μl DNA.

Organism	Genome size bp (10)	CFU/ml	DNA ng/ul (x2)	Purity 260/280	Minimum DNA (ng)	Min CFU	Cq Mean single plex	Cq Mean triplex
*P. aeruginosa*	6901248	6.44E+08	84.60	1.93	0.0012	1.83E+04	17.795	18.55
*B. contaminans*	8711463	5.80E+08	90.40	2.04	0.0015	1.92E+04	19.761	20.492
*M. fujisawaense*	7859068	8.90E+07	9.00	2.24	0.025	4.94E+05	29.056	23.481
*R. insidiosa*	6271672	1.60E+08	43.10	2.04	0.0077	5.72E+04	15.996	18.785
*C. metallidurans*	7278803	3.63E+08	31.40	1.84	0.047	1.09E+06	27.893	40

### 3.3 Quantification of ISS isolates in a polymicrobial community using multiplex qPCR

To assess the performance of species-specific primer sets in a complex microbial mixture, we conducted multiplex qPCR on pooled genomic DNA from five ISS isolates. Due to the limited number of fluorescent detection channels available in the equipment used (Thermo Fisher QuantStudio™ 3), we designed two separate triplex assays: Triplex 1 targeting *P. aeruginosa*, *B. contaminans*, and *M. fujisawaense* (PBM primers); and Triplex 2 targeting *P. aeruginosa*, *R. insidiosa*, and *C. metallidurans* (PRC primers), with *P. aeruginosa* serving as a common reference across both sets. Each reaction was performed using 10 ng/μL total mixed genomic DNA. In Triplex 1, the qPCR successfully detected all three species, with mean Cq values of 18.55 for *P. aeruginosa*, 20.49 for *B. contaminans*, and 23.48 for *M. fujisawaense* (Table 4). In Triplex 2, *P. aeruginosa* and *R. insidiosa* were detected with mean Cq values of 18.55 and 18.79, respectively, while *C. metallidurans* exhibited no amplification (Cq > 40).

**Table 4:** Evaluation of *C. metallidurans* detection in duplex reactions. *C. metallidurans* was run as duplex reactions against the three other species (excluded was *M. fujisawaense* since they share the same TaqMan™ dye). Cq values are based on readings of 10 ng/μl DNA.

	Cq Mean		
Target	Single plex	Duplex	Triplex
*P. aeruginosa*	17.795	19.603	18.55
*B. contaminans*	19.761	21.387	20.492
*M. fujisawaense*	29.056	–	23.481
*R. insidiosa*	15.996	17.683	18.785
*C. metallidurans*	27.893	27.568	>40

To resolve this interference, we conducted three duplex assays excluding the primer set for *M. fujisawaense*, enabling separate detection of *C. metallidurans*. The duplex results revealed Cq values of 19.60 for *P. aeruginosa*, 21.39 for *B. contaminans*, 17.68 for *R. insidiosa*, and 27.57 for *C. metallidurans*, indicating successful and specific amplification for all four targets (Table 4). These results confirm the capability of the designed primer-probe sets to accurately quantify individual bacterial species in a mixed community and validate the utility of the assay for monitoring polymicrobial populations in spaceflight-relevant samples.

### 3.4 Evaluation of protocol in mixed culture samples

As a trial, we tested qPCR detection of mixed culture samples in a standard growth medium, LB broth (Lennox recipe) and in LB containing 100 μg/ml ampicillin, which selectively inhibited *C. metallidurans* (Fig. S2). Following 48h culture, planktonic populations grown in LB (mean log CFU/ml 10.148 ± 0.0198), and LB plus ampicillin (mean log CFU/ml 10.180 ± 0.0232) did not differ significantly (P = 0.362).

Examination of planktonic populations by qPCR is shown in Table 5. As anticipated from the plating experiments, *C. metallidurans* could not be detected by qPCR in planktonic cultures with ampicillin. *M. fujisawaense* was not detected as it was not present in the mixed culture. While biofilm populations did grow (mean log CFU/mm^2^ in LB 5.855 ± 0.158), and LB plus ampicillin (mean log CFU/mm^2^ 5.978 ± 0.0276), there was no significant difference in the biofilm populations (P = 0.520). Initial attempts to extract DNA from biofilm samples were unsuccessful, so that process would need to be optimized in the future. As well in a mixed population biofilm community, some organisms that may be susceptible to an antimicrobial agent in planktonic culture may be protected by other species in a biofilm situation (e.g., Ref. [15]), which emphasizes the importance of monitoring specific population members. While the five organisms used in this study are regularly isolated from the WRS, other organisms have been described (reviewed in Ref. [1]), and complete validation would need to be done in ground-based WRS environments before flight studies are pursued. Employing multiple and alternate gene targets for each organism is certainly an option to enhance specificity or detection limits. As stated earlier, these five organisms represent frequently isolated organisms in the ISS WRS, however many other organisms are present, so specificity and detection limits would need to be tested in this environment.

**Tables 5:** In mixed planktonic culture, all four organisms tested could be detected following growth on LB. Ampicillin inhibited *C. metallidurans* and this organism could not be detected. *M. fujisawaense* was not cultured and was not detected by qPCR. Cq values are shown with standard deviation in parentheses. As described in the text, the populations in the mixed LB and LB plus ampicillin cultures were not significantly different (P = 0.362).

Growth condition	Cq (std dev) LB	Cq (std dev) LB plus ampicillin
*P. aeruginosa*	14.833 (0.566)	14.566 (0.206)
*B. contaminans*	20.508 (0.612)	19.331 (0.172)
*R. insidiosa*	20.950 (0.234)	20.924 (0.217)
*C. metallidurans*	32.196 (1.952)	Not detected
*M. fujisawaense*	Not detected	Not detected

## 4 Conclusions

Based on our findings, all five organisms tested can be detected by single plex qPCR at an estimated detection limit of 10^4^–10^6^ CFU/ml (Table 4). While *C. metallidurans* can be detected and enumerated under duplex qPCR conditions, population estimates of this organism under triplex conditions were less successful. The other four organisms tested, specifically *B. contaminans*, *M. fujisawaense, P. aeruginosa*, and *R. insidiosa* could be tested using multiplex qPCR. Initial testing in a mixed culture showed promise for species detection and changes to one species in the population due to the presence of an antibiotic even though total bacterial populations were not significantly altered (Table 5). Although DNA extraction protocols from biofilms need to be optimized, this study shows the potential for the application of culture-independent monitoring of specific microbial populations in spacecraft and other remote environments without the dependence on specimen transport to reference laboratories. In future missions beyond low Earth orbit, access to reference laboratories will not be possible [16]. The qPCR protocols described in the current study can be adapted in a single plex or multiplex configuration, and the use of a probe (TaqMan chemistry) is recommended to enhance specificity.

## CRediT authorship contribution statement

**Amber Dowell Busboom:** Writing – original draft, Methodology, Investigation, Formal analysis, Data curation, Conceptualization. **Jiseon Yang:** Writing – review & editing, Validation, Software. **Taylor M. Ranson:** Writing – review & editing, Methodology, Investigation. **Cheryl A. Nickerson:** Writing – review & editing, Funding acquisition, Conceptualization. **Evan G. Ortiz:** Software. **Robert J.C. McLean:** Writing – review & editing, Validation, Supervision, Resources, Project administration, Funding acquisition, Conceptualization.

## Competing interests

The authors declare no competing financial interests.

## Data Availability

Data will be made available on request.

## References

[1] Nickerson C.A., McLean R.J.C., Barrila J., Yang J., Thornhill S.G., Banken L.L., Porterfield D.M., Poste G., Pellis N.R., Ott C.M. (2024). Microbiology of human spaceflight: microbial responses to mechanical forces that impact health and habitat sustainability. Microbiology and Molecular Biology Reviews 0.

[2] Pierson D.L., Botkin D.J., Bruce R.J., Castro V.A., Smith M.J., Oubre C.M., Ott C.M., Moldenhauer J. (2012). Environmental monitoring: a comprehensive handbook.

[3] Stahl-Rommel S., Jain M., Nguyen H.N., Arnold R.R., Aunon-Chancellor S.M., Sharp G.M., Castro C.L., John K.K., Juul S., Turner D.J., Stoddart D., Paten B., Akeson M., Burton A.S., Castro-Wallace S.L. (2021). Real-time culture-independent microbial profiling onboard the international space station using nanopore sequencing. Genes.

[4] Castro-Wallace S.L., Chiu C.Y., John K.K., Stahl S.E., Rubins K.H., McIntyre A.B.R., Dworkin J.P., Lupisella M.L., Smith D.J., Botkin D.J., Stephenson T.A., Juul S., Turner D.J., Izquierdo F., Federman S., Stryke D., Somasekar S., Alexander N., Yu G., Mason C.E., Burton A.S. (2017). Nanopore DNA sequencing and genome assembly on the international space station. Sci Rep.

[5] Burton A.S., Stahl S.E., John K.K., Jain M., Juul S., Turner D.J., Harrington E.D., Stoddart D., Paten B., Akeson M., Castro-Wallace S.L. (2020). Off Earth identification of bacterial populations using 16S rDNA nanopore sequencing. Genes.

[6] Khodadad C.L.M., Oubre C.M., Castro V.A., Flint S.M., Roman M.C., Ott C.M., Spern C.J., Hummerick M.E., Maldonado Vazquez G.J., Birmele M.N., Whitlock Q., Scullion M., Flowers C.M., Wheeler R.M., Melendez O. (2021). A microbial monitoring system demonstrated on the international space station provides a successful platform for detection of targeted microorganisms. Life.

[7] Venkateswaran K., Vaishampayan P., Cisneros J., Pierson D.L., Rogers S.O., Perry J. (2014). International space station environmental microbiome - microbial inventories of ISS filter debris. Appl Microbiol Biotechnol.

[8] Zea L., McLean R.J.C., Rook T.A., Angle G., Carter D.L., Delegard A., Denvir A., Gerlach R., Gorti S., McIlwaine D., Nur M., Peyton B., Stewart P.S., Sturman P., Velez Jusiniano Y.A. (2020). Potential biofilm control strategies for extended spaceflight missions. Biofilm.

[9] Carter D.L., Pruitt J.M., Brown C., Bazley J., Gazda D., Schaezler R., Thomas F. (2017). 47th international conference on environmental systems.

[10] Castro C.L., Velez-Justiniano Y.A., Stahl-Rommel S., Nguyen H.N., Almengor A., Dunbar B., McLean R.J.C., Sysoeva T.A., Castro-Wallace S.L. (2023). Genome sequences of bacteria isolated from the international space station water systems. Microbiol Resour Announc.

[11] Mettler M.K., Peyton B.M. (2025). Multimodal biofilm control strategies of spacecraft water systems: evaluating coatings, nutrient removal, and biocides for improved sustainability. Gravitational and Space Research.

[12] Sun J., Lu F., Luo Y., Bie L., Xu L., Wang Y. (2023). OrthoVenn3: an integrated platform for exploring and visualizing orthologous data across genomes. Nucleic Acids Res.

[13] Maurin M. (2012). Real-time PCR as a diagnostic tool for bacterial diseases. Expert Rev Mol Diagn.

[14] Ranson T.M., Barton M.E., McLean R.J.C. (2023). Influence of central metabolism disruption on Escherichia coli biofilm formation. Can J Microbiol.

[15] Whiteley M., Ott J.R., Weaver E.A., McLean R.J.C. (2001). Effects of community composition and growth rate on aquifer biofilm bacteria and their susceptibility to betadine disinfection. Environ Microbiol.

[16] National Academies of Sciences E (2023). Thriving in space: ensuring the future of biological and physical sciences research: a decadal survey for 2023-2032.

