# Supplementary material for: Development of a high-resolution multiplex qPCR method to profile microbial consortia in spaceflight water recovery systems
Source: Biofilm. 2026 Jan 29;11:100349. doi: 10.1016/j.bioflm.2026.100349 (PMC12891862; doi:10.1016/j.bioflm.2026.100349)

**Supplemental Information**

Calibration of OD600 and CFU is shown in Figure S1.

**Figure S1** – Linear regression plot (OD/CFU) of *P. aeruginosa* (P.a.), *B. contaminans* (B.c.), *M. fujisawaense* (M.f.), *R. insidiosa* (R.i.), and *C. metallidurans* (C.m.). The regression coefficients (R^2^ values) are shown.


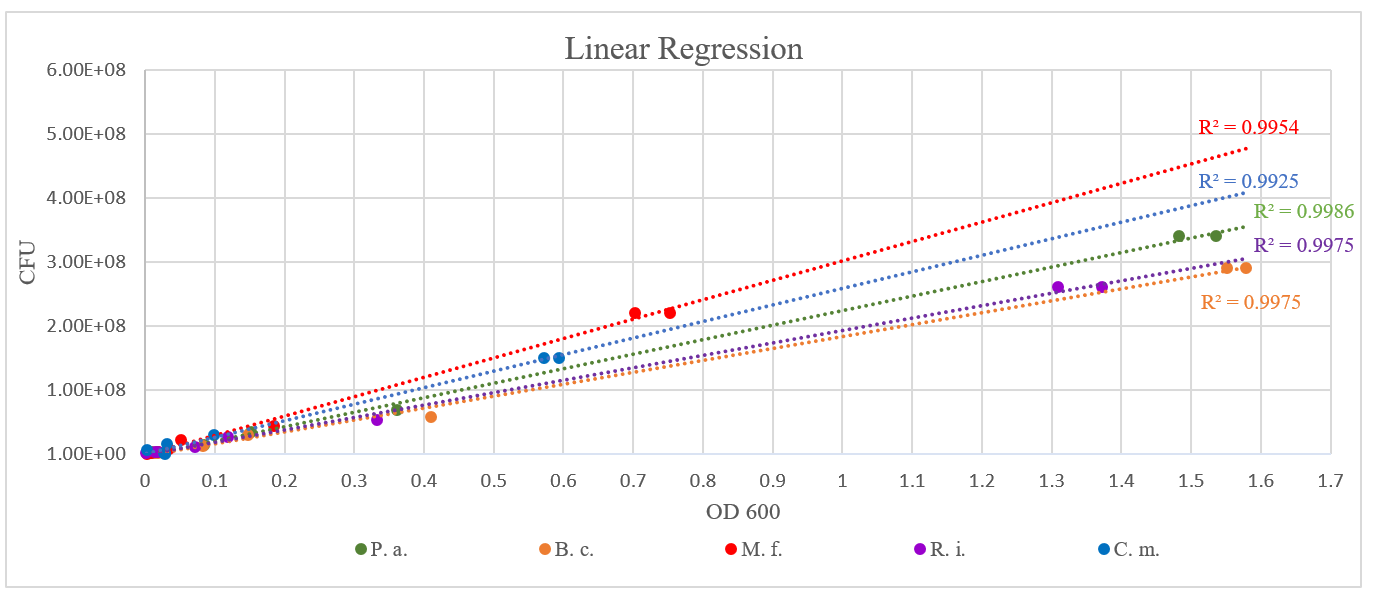


The original and optimized PCR conditions for single plex reactions are shown in **Table S1.** The optimized PCR conditions are also shown in **Table 2**.

**Table S1 –Single plex PCR conditions**

|  | **Original** | | **optimized** | |
| --- | --- | --- | --- | --- |
| hot start | 95°C | 2 min | 95°C | 2 min |
| denature | 95°C | 30 sec | 95°C | 30 sec |
| Anneal | 53.5°C | 30sec | 54.5°C | 15 sec |
| Extension | 72°C | 60 sec | 72°C | 60 sec |
| Final extension | 72°C | 5min | 72°C | 5min |

Species-specific primer sets were designed as described in the Methods section and screened for specificity using PCR simulations in Snapgene. Based on predicted specificity and performance, one primer set per strain was selected for experimental validation (**Table 1**). Each selected primer set was tested by PCR using genomic DNA from all five bacterial strains to evaluate cross-species specificity. Under the original PCR conditions (**Table S1 original conditions**), the primer sets for *P. aeruginosa, B. contaminans,* and *C. metallidurans* yielded single, strain-specific amplicons without detectable off-target amplification (**Fig. S1 panels A, B, and E**). However, the *M. fujisawaense* primer set produced nonspecific bands when tested against genomic DNA from *P. aeruginosa, R. insidiosa*, and *C. metallidurans*, indicating cross-reactivity (**Fig. S1 panel C**). Similarly, the *R. insidiosa* primer set amplified an unexpected ∼400 bp product when tested with *B. contaminans* DNA (**Fig. S1 Panel D**). To improve specificity, the annealing temperature was increased and extension time reduced during PCR optimization as shown (**Table S1 – optimized column** also shown in **Table 2**). These adjustments successfully resulted in specific PCR products at the expected size (**Fig. 1**).

**Figure S1**. Agarose gels show primer specificity testing. (A) P. aeruginosa primers. (B) B. contaminans primers. (C) M. fujisawaense primers. (D) R. insidiosa primers. (E) C. metallidurans primers. Tested against DNA in columns: L= Ladder, 1) P.a., 2) B.c., 3) M.f., 4) R.i., 5) C.m., 6) negative control.


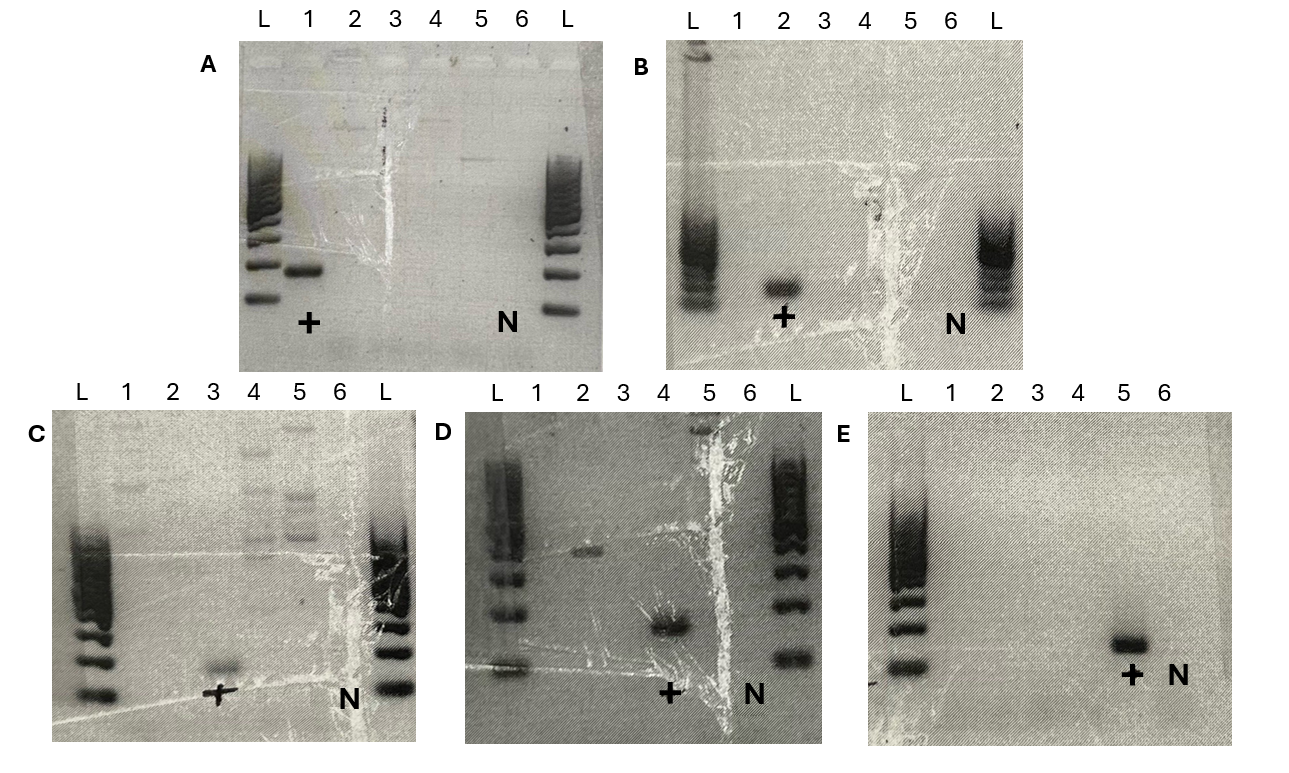


**Evaluation of qPCR in mixed culture** To test the application of qPCR in a mixed culture setting, *B. contaminans,* *C. metallidurans*, *P. aeruginosa,* and *R. insidiosa* were grown in the presence of various antibiotics and disinfectants to see whether a particular treatment could inhibit a subset of these organisms. As shown in **Figure S2**, *C. metallidurans* did not grow in the presence of LB plus 100 µg/ml ampicillin and so that antibiotic was used during the evaluation of qPCR in mixed culture (**Table 5**).

**Figure S2** – Culturing of *B. contaminans,* *C. metallidurans*, *P. aeruginosa,* and *R. insidiosa* on LB plus 100 µg/ml ampicillin showed inhibition of *C. metallidurans*.


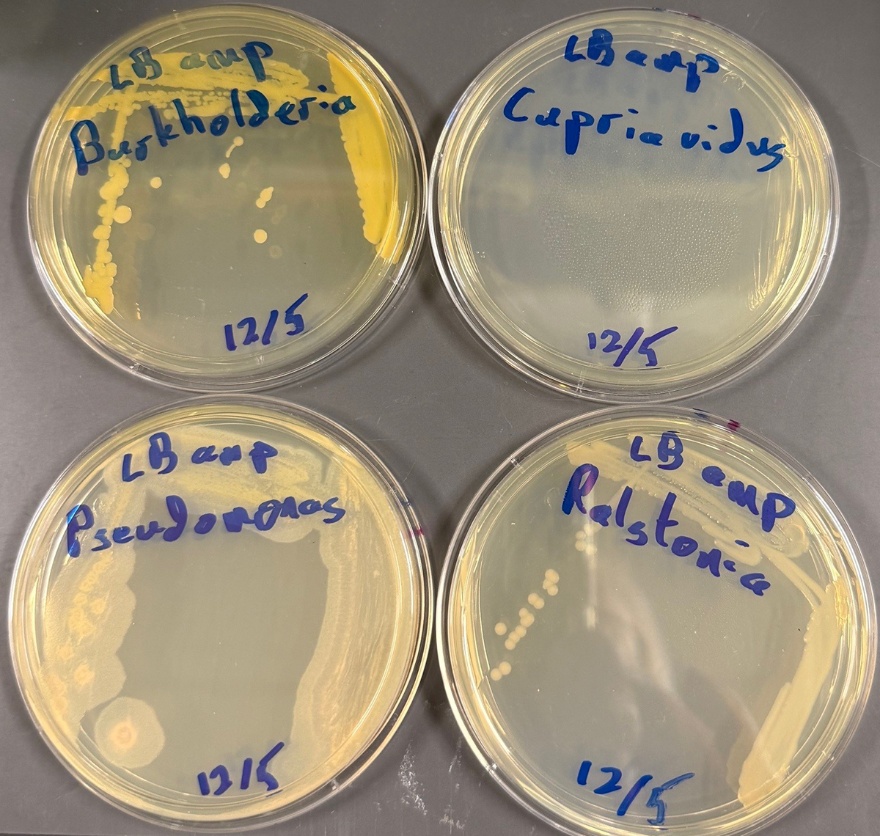

Supplement: Multimedia component 1 [file mmc1.docx]
